# Parental concerns correspond to earliest age of autism diagnosis in increased likelihood infant cohort

**DOI:** 10.3389/frcha.2025.1722543

**Published:** 2026-01-07

**Authors:** Kal Clintberg, Lori-Ann R. Sacrey, Lonnie Zwaigenbaum, Jessica A. Brian, Peter Szatmari, Tracy Vaillancourt

**Affiliations:** 1 Autism Research Centre, Glenrose Rehabilitation Hospital and Department of Pediatrics, University of Alberta, Edmonton, AB, Canada; 2 Bloorview Research Institute and Department of Pediatrics, University of Toronto, Toronto, ON, Canada; 3 Centre for Addiction and Mental Health, Department of Psychiatry, University of Toronto, Toronto, ON, Canada; 4 Counselling Psychology, Faculty of Education, University of Ottawa, Ottawa, ON, Canada

**Keywords:** autism spectrum disorder, infant, longitudinal data, parent concern, sibling study

## Abstract

Autism spectrum disorder (ASD) encompasses a set of behavioural features with a diverse range of presentations, challenges, and trajectories. Previous research has demonstrated how behavioural and developmental markers can differentiate autistic from non-autistic children as early as the first year of life, however there is a dearth of literature demonstrating heterogeneity amongst the clinical presentations of autistic infants. An understanding of the breadth of experiences is necessary to refine and expand early identification methods to identify those currently being overlooked. The current study examined the heterogeneity of early behaviour in a longitudinal cohort of infant siblings of autistic children (*n* = 72) who later received an ASD diagnosis. Parent reports of early behaviour, generated at six time points between six and 24 months of age, describing the presence or absence of concerns across 10 developmental domains, were assessed to compare subgroups of infants based on the earliest age they were diagnosed as autistic (18, 24, or 36 months). The average number of concerns across domains, in addition to the proportion of each subgroup reporting a concern in each of the 10 domains, was compared at each time point. The infants diagnosed at 18 months had a higher average number of concerns across all time points compared to those diagnosed at 24 or 36 months, who demonstrated similar profiles. Play, language, and language regression concerns resulted in the largest effect sizes between groups. These findings indicate that (i) there is heterogeneity in early autism presentations, (ii) within the context of early identification, lack of diagnosis at one age does not eliminate the possibility of future diagnosis, and (iii) that parent reports of early concerns can provide valuable information that can alert clinicians to the features of autism, further attention to which may help reduce disparities in age of diagnosis.

## 1 Introduction

Autism spectrum disorder (ASD) is a neurodevelopmental condition that manifests as restricted and repetitive behaviours (RRBs) and social-communication characteristics which differ from those of non-autistics (1). Although the current average age of diagnosis is approximately 4 years (2), there is evidence that ASD can be detected as early as the first year of age based on attentional differences, brain-based biomarkers, communication and social characteristics, motor delays, RRBs, sensory sensitivity and habituation, as well as trajectories of cognitive and language development (3). Emerging characteristics may present specifically within the RRB diagnostic domain as a need for routines, strong preoccupations with parts of objects, and repetitive or stereotyped movements (stims), and within the social communication domain as less initiation of social interactions and less emotional and social reciprocity than neurotypical infants (4). Earlier identification of ASD comes with the ability to begin interventions sooner, tailored to the child's unique strengths and challenges. Evidence shows that early interventions lead to positive outcomes in cognitive measures, in addition to daily living, motor, language, and social communication skills (5). Autism is a lifelong condition and supports need to extend through adolescence and adulthood to promote quality of life (6). Understanding the heterogeneity of emerging autistic features is therefore critical for improving early identification and intervention, as well as for supporting long-term outcomes.

The data used in this paper are from a longitudinal study that followed children, starting in infancy, who have an older autistic sibling. Such populations have proven to be informative for studying early autism features due to their increased likelihood of being autistic compared to the general population (7). The systematic review by Jones et al. (4) provides an in-depth review of the findings of prospective infant sibling studies, noting that subtle differences can emerge in the first year (in attention, motor, and sensory domains), with social, communication, and RRB differences emerging in the second year. Previous studies of the present cohort have explored the accuracy of various early detection methods (8–11), including prospectively asking parents about their early concerns in infancy to predict ASD diagnosis (12) and the stability of early diagnoses (13). This study extends on the work of our research group, which demonstrated that parent concerns before age two can predict later ASD diagnosis (12), with differences in standardized testing scores differing between subgroups of autistic children based on the earliest age they were diagnosed (13).

The main objective of the current study was to explore differences in parent concerns among infant siblings as grouped by the age of earliest ASD diagnosis. Sacrey et al. (12) demonstrated that parent reports of motor and sensory concerns distinguished between autistic and non-autistic groups in the first year of life, whereas social skills and repetitive behaviours distinguished autistic from non-autistic children during the second year. The pattern of domain differences within the autistic children is unexplored both by age at diagnosis and age of observation. We hypothesized that (i) those diagnosed at 18 months would have the highest number of reported parent concerns at each time observed, (ii) domain-specific relative differences would be present between groups, and (iii) group differences would continue across the ages of observations, indicating phenotypic differences corresponding with earliest age of diagnosis.

## 2 Methods

### 2.1 Procedure

All participants were recruited between 6 and 12 months of age from one of four sites in Canada (Glenrose Rehabilitation Hospital in Edmonton, Alberta; Holland Bloorview Kids Rehab in Toronto, Ontario; IWK Health Centre in Halifax, Nova Scotia; or Offord Centre for Child Studies in Hamilton, Ontario). Recruitment was done by a combination of community self-referral and clinical recruitment by healthcare providers of the older siblings. The research ethics board at each institution approved this study and the caregivers (hereafter, parents) of the infant siblings provided written informed consent prior to participation in the study.

### 2.2 Participants

The subset of the longitudinal cohort used in this study was made up of the 72 infant siblings who were diagnosed with ASD by age three who have data available for a minimum of one parent concern report. A multidisciplinary team confirmed the diagnosis of the older siblings using DSM-IV-TR criteria. The infant siblings and probands were confirmed to understand English, were born between 36 and 42 weeks gestation, with a birth weight exceeding 2,500 grams, and had no known genetic or neurological conditions nor severe motor or sensory impairments.

### 2.3 Parent concerns data

Parent concerns data were collected using a semi-structured interview, which assessed 10 developmental domains – behaviour, diet, language, language regression, motor, play, RRBs, sensory, sleep, and social – which can be more broadly described within the categories of behavioural, communication, and general concerns. Sacrey et al. (12) digitally transcribed data from paper copies of the interviews into Microsoft Excel (Version 14.4.1), removed identifying information, and replaced it with a unique case number. A researcher who was blinded to group membership then coded whether the parent described a concern for each domain in a binary format, with “0” representing the absence of a concern, including if the domain was left blank, or “1” to indicate a concern present. A second researcher then coded 30% of the interviews, and when analyzed using Cohen's *K*, demonstrated interrater reliability of 0.71 (12).

### 2.4 Diagnostic assessment

Diagnoses were made at the 18- and 24-month visits based on developmental history, informed by the parent concerns interview, Autism Diagnostic Observation Schedule (ADOS), and additional standardized testing, while diagnoses established at the 36- to 42-month age visit (hereafter referred to as the 36-month visit) were based on a clinical best estimate decision informed by the Autism Diagnostic Interview-Revised (ADI-R), ADOS, and DSM-IV-TR. For a complete list of measures used, see Zwaigenbaum et al. (13). Assessments were completed by child psychiatrists, clinical psychologists, and developmental pediatricians, each with a minimum of 10 years of diagnostic experience with ASD (13). To allow for between-group comparisons, participants were stratified and grouped based on the earliest age at which they received a diagnosis of ASD (first diagnosis (FD) at the 18-month (FD-18), 24-month (FD-24), or 36-month (FD-36) visit).

### 2.5 Assessment measures

#### 2.5.1 Parent concern reports

Sacrey et al. (12) developed an interview to collect information about parent concerns in infants from 6 to 24 months old. The interview is semi-structured, with the interviewer first asking parents broadly if they have any current questions or concerns, followed by specific questions covering 10 developmental domains in a standardized order (see Supplementary Material for the interview).

#### 2.5.2 Autism Diagnostic Interview-Revised (ADI-R)

The ADI-R is a standardized semi-structured interview used to gather information regarding an individual's communication and language skills, reciprocity in social interactions, and repetitive behaviours and interests. It is validated for use beginning at 18 months and extending into adulthood. Scores from the ADI-R can be used to inform an autism diagnosis using DSM-IV or ICD-10 criteria, plan treatment, and distinguish autism from other developmental disorders (14).

#### 2.5.3 Autism Diagnostic Observation Schedule (ADOS)

The ADOS is a semi-structured direct interactive measure used to assess communication, imaginative play, social reciprocity, and repetitive behaviours. It contains four modules, which are each appropriate for a different level of language abilities and/or age. The evaluation leads to subscale scores in communication, play, repetitive behaviour, and social domains. The communication and social domain scores are used in diagnosing autism, and differentiation from other developmental disorders (15).

### 2.6 Statistical analysis

Statistical analyses were performed using the Statistical Package for the Social Sciences (SPSS) version 29 to compare the FD-18, FD-24, and FD-36 groups. Supplemental calculations were performed using Microsoft Excel (Version 2410).

First, Chi-square tests were performed to compare assigned sex at birth, maternal race, paternal race, and data missingness. Parent races were collected and recorded based on open-ended self-reports and later binary coded as “non-racialized” for those who self-identified as Caucasian, white, or of European descent, or “racialized” for those of non-European descent. One-way ANOVAs were used to assess for between-group differences in family socioeconomic status (SES), indexed using the Hollingshead Four Factor Index, the child's age at their first visit, as well as exact age at the 18-, 24-, and 36-month assessments.

The data from the parent concern forms were assessed in three ways. First, the sum of concerns across all domains was calculated for each child and at each age of observation with a report available by tallying the number of domains with a concern reported (coded as “1”) at the given age, resulting in a score between 0 and 10. Secondly, proportions of concerns reported across all time points and within a specified domain were calculated for each child by tallying the number of concerns for the given domain at each of the 6 ages of observation, divided by the total number of reports available for that domain (any cell with a non-missing value) to compensate for variation among participants in the number of parent concern reports completed. This resulted in a percentage between 0% and 100% indicating the frequency of reports with a concern in each domain. Lastly Chi-square tests of independence were performed comparing the FD-18, FD-24, and FD-36 groups at each domain-time pairing. In cases where greater than 20% of the cells had an expected count of less than 5, a Fisher exact test (2 × c) was conducted to assess significance. Planned comparisons on significant group differences were subjected to Bonferroni corrections to provide a conservative estimate of sub-group relationships. Further, effect sizes were explored using Cramer's V and interpreted using the guidelines described by IBM (16); small: ES ≤ 0.2, moderate: 0.2 < ES ≤ 0.6, strong: ES > 0.6.

The concerns data were not normally distributed, as determined by the Shapiro–Wilk test (17), and lacked consistent homogeneity of variance, as assessed by Levene's test (18). Accordingly, Kruskal–Wallis H non-parametric test was selected to compare the sum of concerns at each observation age and the proportion of concerns within each domain. As above, *post hoc* analysis was conducted on models with significance at the α = 0.05 level using Dunn's (19) pairwise comparisons procedure, with Bonferroni corrections for multiple comparisons to identify specific group associations.

## 3 Results

### 3.1 Demographics and characteristics

Assigned sex at birth was not equally represented; 52 of the 72 infants (72.2%) were assigned male. A Chi-square test of independence revealed no significant relation between earliest age of diagnosis and assigned sex (χ^2^(2) = 0.603, *p* = 0.740). There was a higher representation of non-racialized than racialized parents, with 55 out of 71 fathers (77.5%) and 48 out of 70 mothers (68.6%) identifying as non-racialized. There was no significant group difference for paternal (χ^2^(2) = 0.364, *p* = 0.834) or maternal race (χ^2^(2) = 1.606, *p* = 0.448). There were no significant differences for missing reports between the subgroups at any age of observation (6-months: χ^2^(2) = 2.843, *p* = 0.241; 9-months: χ^2^(2) = 1.043, *p* = 0.600; 12-months: χ^2^(2) = 2.580, *p* = 0.275; 15-months: χ^2^(2) = 1.911, *p* = 0.385; 18-months: χ^2^(2) = 2.426, *p* = 0.297; 24-months: χ^2^(2) = 1.182, *p* = 0.554).

One-way ANOVA indicated no statistically significant differences between the FD-18, FD-24, and FD-36 groups for family SES (*F*(2, 68) = 0.518, *p* = 0.598), age at the first visit (*n* = 72, *F*(2, 69) = 1.138, *p* = 0.326), or exact age at the 18-month (*n* = 69, *F*(2, 66) = 1.646, *p* = 0.201), 24-month (*n* = 68, *F*(2, 65) = 0.191, *p* = 0.826), or 36-month (*n* = 70, *F*(2, 67) = 0.909, *p* = 0.408) time points. Demographic characteristics are described in Table 1 and the number of reports available by subgroup at each time point are reported in Table 2.

**Table 1:** Participant characteristics.

Characteristic	Timing of diagnosis						Total		*Χ*^2^	*p*-value
	18-month visit		24-month visit		36-month visit					
	*n* = 11		*n* = 30		*n* = 31		*n* = 72			
Sex (F:M)	2:9	(1:4.5)	9:21	(1:2.3)	9:22	(1:2.4)	20:52	1:2.6	0.603	.740
Maternal ethnicity (racialized:non-racialized)	4:7	(1:1.8)	11:18	(1:1.6)	7:23	(1:3.3)	22:48	1:2.2	1.606	.448
Paternal ethnicity (racialized:non-racialized)	3:8	(1:2.7)	7:22	(1:3.1)	6:25	(1:4.2)	16:55	1:3.4	0.364	.834
	Mean	SD	Mean	SD	Mean	SD	Mean	SD	*F*-test	*p*-value
Family SES^a^	47.1	(15.3)	43.7	(14.0)	47.1	(13.6)	45.7	(13.9)	0.518	.598
Age at first visit^b^	8.3	(2.5)	8.4	(2.5)	9.3	(2.8)	8.8	(2.6)	1.138	.326
Age at 18-month visit^b^	18.4	(0.2)	18.6	(0.7)	18.4	(0.4)	18.5	(0.5)	1.646	.201
Age at 24-month visit^b^	24.6	(0.7)	24.5	(0.6)	24.5	(0.5)	24.5	(0.5)	0.191	.826
Age at 36-month visit^b^	40.4	(4.0)	39.3	(3.1)	38.8	(3.1)	39.3	(3.3)	0.909	.408

a Based on the Hollingshead Index.

b Given in months.

**Table 2:** Number of parent concern reports by age of report and earliest diagnosis.

Age of report	Timing of diagnosis			Total (*n*)
	18-months (*n*)	24-months (*n*)	36-months (*n*)	
Group size	11	30	31	72
6-month report	6	8	10	24
9-month report	7	14	17	38
12-month report	10	23	28	61
15-month report	9	20	25	54
18-month report	11	25	25	61
24-month report	11	27	28	66

### 3.2 Sums of concerns

A Kruskal–Wallis H assessed group differences between the average number of concerns reported for children at each of the 6 ages of observations. Mean concerns reported did not differ by groups at the 6-month (*H*(2) = 4.058, *p* = 0.131) or 9-month (*H*(2) = 3.934, *p* = 0.140) time points, but did differ at the 12-month (*H*(2) = 6.637, *p* = 0.036), 15-month (*H*(2) = 7.205, *p* = 0.027), 18-month (*H*(2) = 8.622, *p* = 0.013), and 24-month (*H*(2) = 10.029, *p* = 0.007) time points. Bonferroni *post hoc* analyses identified differences between the FD-18 and FD-36 groups at each time point between 12- and 24-months (12-month, *p* = 0.032; 15-month, *p* = 0.022; 18-month, *p* = 0.011; 24-month, *p* = 0.005), but comparisons between FD-24 and FD-18 nor FD-36 groups were not different at any time point measured. For this and all following described *post hoc* analysis results, the FD-18 group was found to have a higher number of concerns than the later diagnosed group.

A Kruskal–Wallis H assessed group differences for the average proportion of reports with a concern for a given domain, across all 6 time points combined. Group differences were not significant in the behaviour (*H*(2) = 4.742, *p* = 0.093), diet (*H*(2) = 1.277, *p* = 0.528), motor (*H*(2) = 1.704, *p* = 0.426), RRB (*H*(2) = 2.033, *p* = 0.362), sensory (*H*(2) = 5.615, *p* = 0.060), or social (*H*(2) = 1.809, *p* = 0.405) domains. The language (*H*(2) = 8.877, *p* = 0.012) and sleep (*H*(2) = 6.547, *p* = 0.038) domains demonstrated significant group differences, with Bonferroni *post hoc* comparisons resulting in differences between the FD-18 and FD-36 groups (language, *p* = 0.009; sleep, *p* = 0.037), but not between the FD-24 and FD-18 or FD-36 groups. The language regression (*H*(2) = 8.063, *p* = 0.018) and play (*H*(2) = 13.717, *p* = 0.001) domains were different between group, with significant *post hoc* comparisons between the FD-18 and FD-24 groups (language regression, *p* = 0.049; play, *p* = 0.001) and the FD-18 and FD-36 groups (language regression, *p* = 0.016; play, *p* = 0.016), but not between the FD-24 and FD-36 groups.

The language domain had the highest frequency of concern for each group (FD-18 mean ± SD = 77.7% ± 22.4%; FD-24 mean ± SD = 49.6% ± 32.6%; FD-36 mean ± SD = 42.0% ± 35.9%) and across all groups combined (mean ± SD = 50.6% ± 34.6%). Language regression had the lowest frequency of complaints across groups (mean ± SD = 11.3% ± 18.4%). A full description of the frequency of concerns across all ages of observation is shown in Table 3.

**Table 3:** Domains ranked by relative frequency of complaints.

Domain rank	Timing of diagnosis						Total	
	18-month visit		24-month visit		36-month visit			
	Domain	Mean ± SD (%)	Domain	Mean ± SD (%)	Domain	Mean ± SD (%)	Domain	Mean ± SD (%)
1	Language	77.7 ± (22.4)	Language	49.6 ± (32.6)	Language	42.0 ± (35.9)	Language	50.6 ± (34.6)
2	Sensory	59.2 ± (33.0)	Behaviour	42.4 ± (39.5)	Motor	36.0 ± (35.6)	Behaviour	39.9 ± (35.3)
3	Behaviour	57.3 ± (28.0)	Motor	36.4 ± (33.9)	Social	34.8 ± (31.1)	Motor	38.3 ± (34.6)
4	Motor	50.0 ± (34.1)	Sensory	34.7 ± (32.5)	Behaviour	31.2 ± (31.5)	Sensory	37.0 ± (33.3)
5	Sleep	50.0 ± (30.0)	Social	34.6 ± (36.1)	Sensory	31.2 ± (31.8)	Social	36.0 ± (32.1)
6	Play	47.0 ± (32.3)	RRBs	33.2 ± (38.1)	RRBs	25.9 ± (33.1)	RRBs	31.3 ± (35.6)
7	Social	43.3 ± (23.6)	Sleep	28.7 ± (37.3)	Sleep	22.0 ± (28.2)	Sleep	29.0 ± (33.4)
8	RRBs	41.2 ± (35.8)	Diet	27.9 ± (34.0)	Diet	18.7 ± (27.4)	Diet	23.3 ± (30.2)
9	Language regression	28.2 ± (26.1)	Play	11.7 ± (28.1)	Play	16.0 ± (23.7)	Play	19.0 ± (29.2)
10	Diet	24.1 ± (27.4)	Language regression	9.9 ± (17.1)	Language regression	6.70 ± (12.8)	Language regression	11.3 ± (18.4)

### 3.3 Specific age-domain concern differences

**Figure 1:**
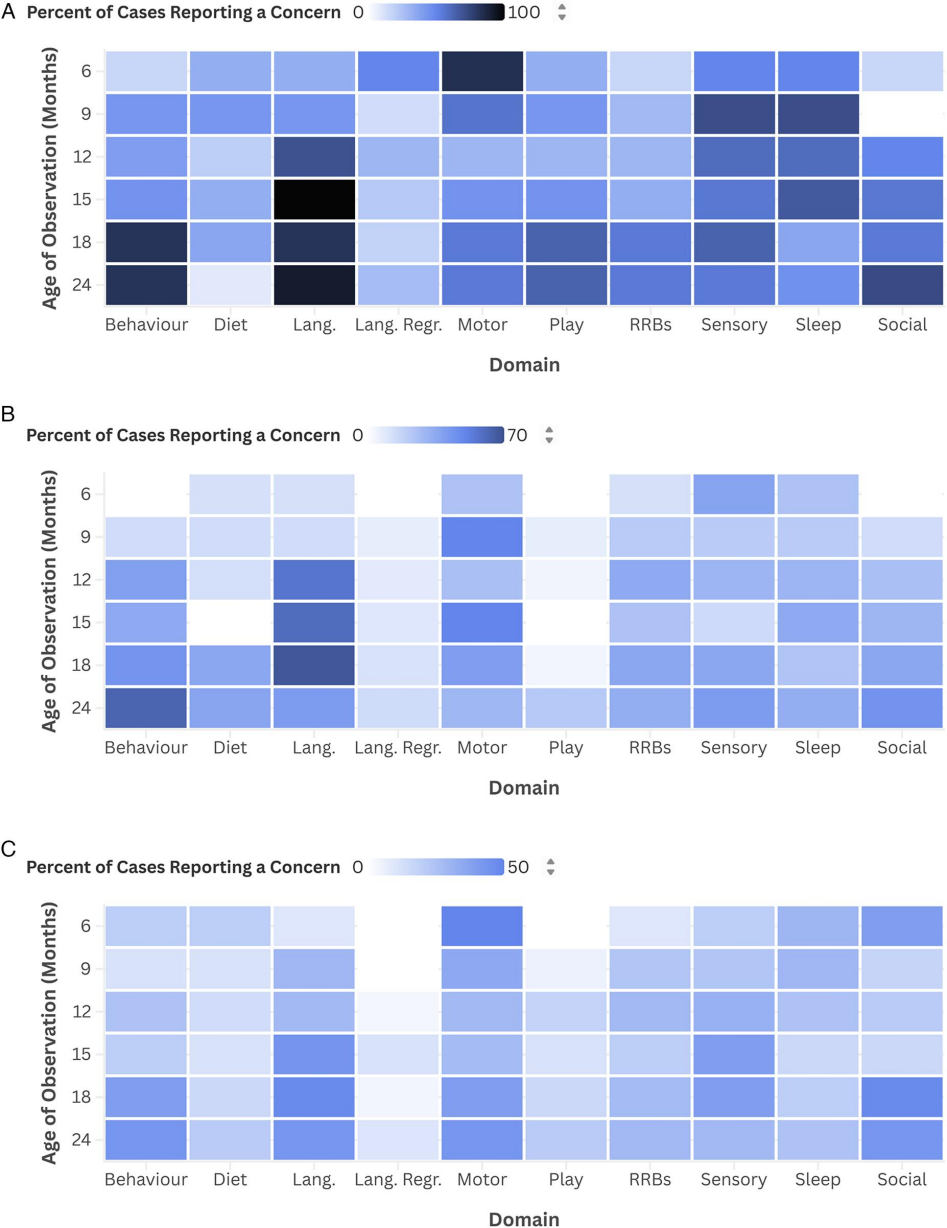
Proportion of subgroup with a concern at each domain-time pairing. **(A)** FD-18; **(B)** FD-24; **(C)** FD-36.

Chi-square tests of independence were performed on all 60 domain-time pairings, but only the effect sizes and significant results are presented below. Complete results, including directions of *post hoc* analyses, are found in Tables 4, 5, and in Figure 1 which depicts the percentage of each subgroup who reported a concern at each domain-time pairing (with Figures 1A–C corresponding to data from the FD-18, -24, and -36 groups, respectively). The 6- (*n* = 24) and 9-month (*n* = 38) time points had the fewest respondents, limiting statistical power. Time points between 12 and 24 months had between 54 and 66 respondents.

**Table 4:** Descriptives and Kruskal–Wallis test results for sum of concerns at each report and proportion for each domain.

	Timing of diagnosis						Total		*H*	*p*-value	Post hoc^c^
	a		b		c						
	18-month visit		24-month visit		36-month visit						
	Mean	(SD)	Mean	(SD)	Mean	(SD)	Mean	(SD)			
Time of report^a^											
6-month	3.83	(2.86)	1.25	(1.39)	2.00	(1.49)	2.21	(2.06)	4.058	.131	
9-month	4.14	(3.24)	1.86	(1.75)	1.88	(1.97)	2.29	(2.29)	3.934	.140	
12-month	4.20	(1.99)	2.70	(2.12)	2.25	(1.67)	2.74	(1.99)	6.637	.036*	a > c
15-month	5.00	(3.08)	2.60	(1.50)	2.20	(1.76)	2.81	(2.16)	7.205	.027*	a > c
18-month	5.45	(2.46)	3.36	(1.87)	3.00	(2.23)	3.59	(2.28)	8.622	.013*	a > c
24-month	5.55	(1.75)	3.59	(2.10)	3.07	(2.34)	3.70	(2.29)	10.029	.007*	a > c
	Mean (%)	(SD; %)	Mean (%)	(SD; %)	Mean (%)	(SD; %)	Mean (%)	(SD; %)	***H***	***p*-value**	Post hoc*
Domain^b^											
Behaviour	57.3	(28.0)	42.4	(39.5)	31.2	(31.5)	39.9	(35.3)	4.742	.093	
Diet	24.1	(27.4)	27.9	(34.0)	18.7	(27.4)	23.3	(30.2)	1.277	.528	
Language	77.7	(22.4)	49.6	(32.6)	42.0	(35.9)	50.6	(34.6)	8.877	.012*	a > c
Language regression	28.2	(26.1)	9.9	(17.1)	6.7	(12.8)	11.3	(18.4)	8.063	.018*	a > b,c
Motor	50.0	(34.1)	36.4	(33.9)	36.0	(35.6)	38.3	(34.6)	1.704	.426	
Play	47.0	(32.3)	11.7	(28.1)	16.0	(23.7)	19.0	(29.2)	13.717	.001*	a > b,c
RRBs	41.2	(35.8)	33.2	(38.1)	25.9	(33.1)	31.3	(35.6)	2.033	.362	
Sensory	59.2	(33.0)	34.7	(32.5)	31.2	(31.8)	37.0	(33.3)	5.615	.060	
Sleep	50.0	(30.0)	28.7	(37.3)	22.0	(28.2)	29.0	(33.4)	6.547	.038*	a > c
Social	43.3	(23.6)	34.6	(36.1)	34.8	(31.1)	36.0	(32.1)	1.809	.405	

a Calculated as the sum of domains with a concern reported at the given time of report.

b Domain sums calculated as the sum of concerns for the given domain across all times of reports, divided by the total number of reports completed.

c Post hoc analysis based on pairwise comparisons with Bonferroni corrections for multiple comparisons.

* Significant at *α* = 0.05.

**Table 5:** Chi-Square results by age of report and domain.

Domain	*χ* ^2^	Cramer’s V	*p*-value^a^	Post hoc^b^	Domain	*χ* ^2^	Cramer’s V	*p*-value^a^	Post hoc^b^
6-month report					15-month report				
Behaviour	1.752^c^	.270	.416		Behaviour	2.336	.208	.311	
Diet	0.909^c^	.195	.635		Diet	7.020^c^	.361	.030*	a > b
Language	1.620^c^	.260	.445		Language	8.603	.399	.014*	a > c
Language regression	10.286^c^	.655	.006*	a > c	Language regression	0.860^c^	.126	.650	
Motor	4.667^c^	.441	.097		Motor	2.403	.211	.301	
Play	6.545^d^	.522	.054^d^		Play	10.905^d^	.449	.004*	a > b
RRBs	0.152^c^	.080	.927		RRBs	0.659^c^	.110	.719	
Sensory	1.613^c^	.259	.447		Sensory	5.525	.320	.063	
Sleep	1.050^c^	.209	.592		Sleep	8.058	.386	.018*	a > c
Social	4.396^c^	.428	.111		Social	5.239	.311	.073	
9-month report					18-month report				
Behaviour	3.442^c^	.301	.179		Behaviour	5.800	.308	.055	
Diet	3.442^c^	.301	.179		Diet	2.969	.221	.227	
Language	2.117^c^	.236	.347		Language	4.306	.226	.116	
Language regression	2.187^c^	.240	.335		Language regression	1.956^c^	.179	.376	
Motor	1.206^c^	.178	.547		Motor	0.780	.113	.677	
Play	6.634^d^	.418	.080^d^		Play	17.554^c^	.536	<.001*	a > b,c
RRBs	0.132^c^	.059	.936		RRBs	2.335	.196	.311	
Sensory	6.322^d^	.408	.070^d^		Sensory	2.505	.203	.286	
Sleep	5.498^c^	.380	.064		Sleep	1.111	.135	.574	
Social	1.376^c^	.190	.503		Social	1.305	.146	.521	
12-month report					24-month report				
Behaviour	1.427	.153	.490		Behaviour	5.451	.287	.066	
Diet	0.227^c^	.067	.871		Diet	3.669	.236	.160	
Language	6.770	.333	.034*		Language	8.861	.336	.012*	a > b,c
Language regression	5.857^c^	.310	.053		Language regression	1.688^c^	.160	.430	
Motor	0.066	.033	.968		Motor	2.277	.186	.320	
Play	4.043^c^	.257	.132		Play	7.824	.344	.020*	a > b,c
RRBs	0.234	.062	.889		RRBs	2.393	.190	.302	
Sensory	2.987	.221	.225		Sensory	2.423	.192	.298	
Sleep	4.189	.262	.123		Sleep	1.571	.154	.456	
Social	3.051	.224	.218		Social	3.120	.217	.210	

a Asymptotic 2-sided significance given unless otherwise specified.

b Post hoc analyses performed with multiple z-tests of 2 proportions and Bonferroni corrections for multiple comparisons (a = FD-18; b = FD-24; c = FD-36).

c Greater than 20% of cells have an expected count of less than 5, however, significance was confirmed using Fisher's exact test (2 × c).

d Greater than 20% of cells have an expected count of less than 5, and only the results of the Chi-square test, but not the Fisher exact test, are statistically significant. Exact 2-sided significance reported instead of asymptotic.

* Significant at *α* = 0.05.

The only domain with group differences at 6 months was language regression (χ^2^(2) = 10.286, *p* = 0.006). *Post hoc* analyses indicated differences between the FD-18 and FD-36 groups. At 6 months, the effect sizes were strong for language regression (0.655), moderate for play (0.522), motor (0.441), social (0.428), behaviour (0.270), language (0.260), sensory (0.259), and sleep (0.209), and weak for diet (0.195) and RRBs (0.080).

At 9-months, none of the domains measured by Fisher's Exact test resulted in group differences (all *p*'s > 0.050). Effect sizes were moderate for play (0.418), sensory (0.408), sleep (0.380), behaviour (0.301), diet (0.301), language regression (0.240), and language (0.236), and weak for social (0.190), motor (0.178), and RRBs (0.059).

From the 12-month reports, the language domain revealed between-group differences (χ^2^(2) = 6.770, *p* = 0.034), but *post hoc* analyses did not identify significant pairwise comparisons. Effect sizes were moderate for language (0.333), language regression (0.310), sleep (0.262), play (0.257), social (0.224), and sensory (0.221), and weak for behaviour (0.153), diet (0.067), RRBs (0.062), and motor (0.033).

The diet (Fisher's Exact = 6.322, *p* = 0.037), language (χ^2^(2) = 8.603, *p* = 0.014), play (Fisher's Exact = 9.096, *p* = 0.004), and sleep (χ^2^(2) = 8.058, *p* = 0.018) domains were significant at 15 months. *Post hoc* analyses demonstrated significant differences between the FD-18 and FD-24 groups for diet and play and between FD-18 and FD-36 for language and sleep. Effect sizes were moderate for play (0.449), language (0.399), sleep (0.386), diet (0.361), sensory (0.320), social (0.311), motor (0.211), and behaviour (0.208), and weak for language regression (0.126) and RRBs (0.110).

Only the play domain returned significant differences from the 18-month reports using Fisher's Exact test (Fisher's Exact = 14.735, *p* < 0.001). *Post hoc* analyses determined significant differences between FD-18 and each of FD-24 and FD-36, but not between FD-24 and FD-36. Effect sizes were moderate for play (0.536), behaviour (0.308), language (0.226), diet (0.221), and sensory (0.203), and weak for RRBs (0.196), language regression (0.179), social (0.146), sleep (0.135), and motor (0.113).

From the 24-month reports, the language (χ^2^(2) = 8.861, *p* = 0.012) and play (χ^2^(2) = 7.824, *p* = 0.020) domains demonstrated between-group differences. *Post hoc* analyses determined significant differences between FD-18 and each of the FD-24 and FD-36 groups, but not between FD-24 and FD-36. Effect sizes were moderate for play (0.344), language (0.336), behaviour (0.287), diet (0.236), and social (0.217), and weak for sensory (0.192), RRBs (0.190), motor (0.186), language regression (0.160), and sleep (0.154).

**Figure 2:**
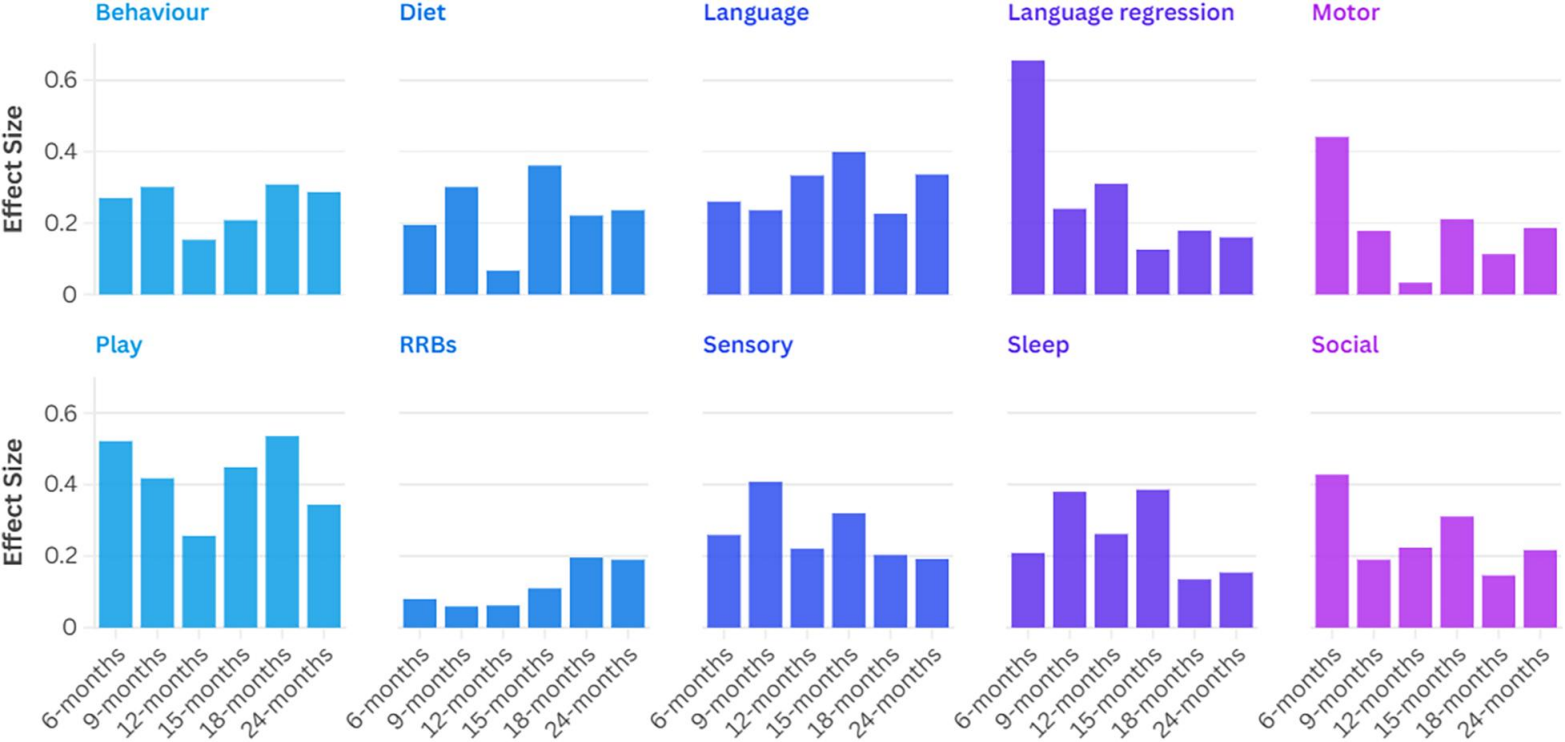
Effect sizes by domain and age of observation.

Overall, the play and language domains had the highest number of between-group differences, each significant at three ages of observation. Diet, language regression, and sleep had significant group differences at one age of report, while the other five domains were not statistically significant at any time. The play domain contributed the most to effect size when considered across all ages of observation, while the RRB and motor domains had the smallest effect. Effect sizes for each domain and age of report are depicted in Figure 2.

## 4 Discussion

This study was the first to explore heterogeneity in infant presentations of autism through concerns reported by parents, comparing the number and nature of concerns at multiple stages of early development and domain-specificity of differences between groups based on earliest age of autism diagnosis. A higher number of total concerns was reported by parents of the group of infant siblings who were diagnosed at 18 months, the youngest age of diagnosis in this study. Further, effect sizes visually varied between the domains measured, indicating unique concern patterns for the youngest diagnosed group compared to those who were diagnosed at age 2 or 3 years. These findings support the existence of unique phenotypes in emerging autism trajectories, as well as the clinical relevance of parent observations.

As predicted, the domains and frequencies of parent concerns differed based on the age of first ASD diagnosis, aligning with the findings of Zwaigenbaum et al. (13) and expanding on Sacrey et al. (12). Differences emerged between children diagnosed at 18 months compared to those diagnosed later (at 24 or 36 months) and were found as early as 6-months, where notably half of the parents of children diagnosed at 18 months reported a concern in language regression, which was not noted as a concern at that age for any infant in the other two subgroups. Our interview used the term “language regression” at this age to describe infants who had begun using any communicative strategies, such as babbling or gesturing, but later stopped. This contrasts with recent meta-analysis of autistic regression describing the average onset of regression as approximately 20 months of age (20). Qualitative review of the concerns data additionally revealed that all 11 infants diagnosed at 18 months reported delayed or absence of words on at least one parent report, compared to 15 of the 30 diagnosed at 24 months and 19 of the 31 diagnosed at 36 months. Additional qualitative data regarding specific concerns can be found in the Supplementary Materials. These unique findings exemplify the variability in clinical presentations between phenotypic subgroups, and the value of research which approaches ASD as a heterogenous condition instead of a monolith.

**Figure 3:**
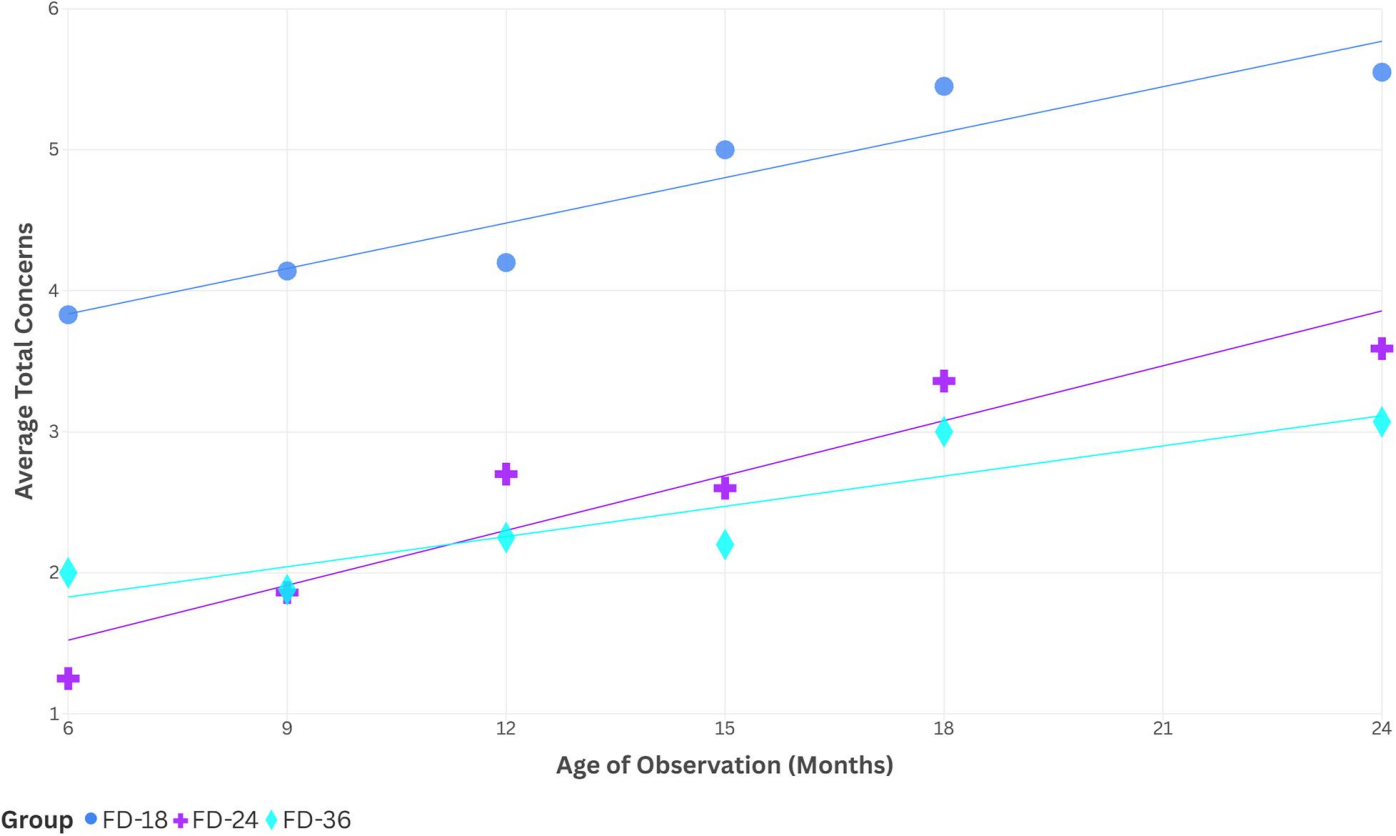
Average number of concerns by subgroup and age of report.

As predicted, the sum of total concerns reported at each age differed between the children diagnosed at 18 months and those diagnosed at 36 months of age. Visual inspection of concerns (see Figure 3) demonstrates an approximately linear increase in concerns with each successive report, with very similar scores for the groups diagnosed at 24 and 36 months. The pattern of parent concern suggests that diagnostic groups may be phenotypically differentiated as “earlier identified” (FD-18) and “later identified” (FD-24 and FD-36 combined). Future research should explore if early parent concerns differ in children who are first diagnosed beyond the age of three.

Between-group differences varied by domain, as expected, with play, language, and language regression domains as the most predictive of which autistic subgroup the child was in, in terms of cumulative effect size. The domains with the largest effect sizes differed from those reported in Sacrey et al. (12), who compared parent concerns of autistic and non-autistic infants, and identified the motor and sensory domains as having the greatest effect sizes in the first year of life, and repetitive behaviours and social skills in the second year. In comparison, the present study identified the motor domain as having the second lowest effect size overall out of the ten domains, with moderate effect sizes at 6 and 15 months and weak effect sizes at other time points. The sensory domain demonstrated moderate effect sizes for group differences at each age, except for 24 months (weak effect). Importantly, that the groups did not differ on motor and sensory concerns was not due to a lack of reported concerns in these domains. Instead, motor and sensory were, respectively, the third and fourth most frequent domains for concerns across all groups; the lack of significance is likely due to a relative homogeneity in the frequency of motor and sensory concerns reported for each group.

Differences in parent concerns regarding play behaviours showed the largest effect size, especially at 15, 18, and 24 months. The play domain was found by Sacrey et al. (12) to differentiate autistic and non-autistic groups but was not identified as a key predictor of diagnostic group. The differences between Sacrey et al. (12) and the present study regarding domain effect sizes suggest that characteristics that distinguish between autistic and non-autistic individuals in infancy differ from those that differentiate subgroups of the autistic population from each other, and highlights the importance of longitudinal analyses such as those used here and in Zwaigenbaum et al. (13). Advancements in screening and diagnostic methods would benefit from considering heterogeneity across development by evaluating subgroups of individuals within the autistic population rather than focusing exclusively on comparing autistics to non-autistics. In the context of intervention, this is consistent with efforts to identify and provide individualized supports and personalized treatment plans.

### 4.1 Limitations

The primary limitation of this study was the small sample, particularly when stratified into subgroups based on age at diagnosis, and for the earliest time points. Although steps were taken to ensure statistical validity (e.g., applying non-parametric tests, including the conservative Fisher's exact test, and Bonferroni corrections on planned comparisons), these choices limited the ability to detect significant differences at the 0.05 level. A larger sample might have uncovered additional subgroup differences, including possibly between children diagnosed at 24 vs. 36 months. Between-group differences can be identified with the present data through visual inspection, such as social concerns at 6 months where 40% of those diagnosed at 36 months reported a concern but none were reported by those diagnosed at 24 months, however, this and other differences did not demonstrate significance. Second, the groups identified are not homogenous, as only the age of first identification, but not the stability of diagnosis, was considered. For three of the infants given a diagnosis at 18 or 24 months, the diagnosis was not confirmed at 36 months. However, in two of these cases (for one infant diagnosed at 18 months and one at 24 months), they were noted by examiners at 36 months to be non-typically developing, as described by Chawarska et al. (21), due to ADOS severity scores of ≥3. The third infant, diagnosed first at 24 months, was noted at 36 months to demonstrate challenges related to ADHD. These cases were retained in analysis because, as the current study demonstrates, lack of identification as autistic at a given time in early childhood does not preclude later diagnosis. Thirdly, this study relied solely on the previous coding of concerns by Sacrey et al. (12) and did not systematically explore the contents of the parent concern interviews in a qualitative manner. A more qualitative approach to analysis of the original interviews may enhance the nuance of the findings and potentially allow for identification of more specific concerns differentiating the subgroups. Fourth, the infants in this study all have an older autistic sibling, meaning the parents' concerns and perspectives may be influenced from experiences with their older child. Further research is required to assess the use of parent concerns as a clinical tool with first-time parents or those with exclusively non-autistic older children. Lastly, the sample is majority male and white, thus generalizability to the larger ASD population is limited and highlights the impact of differential access to resources when it comes to the disparities in autism referrals, diagnoses, and supports received by minority communities (22–24). It should be noted that the binary grouping of racialized and non-racialized individuals is an oversimplification of lived experience, as it overlooks specific identities. Race was not central to the main research objective, and the sample size lacked the statistical power for further between-group classification, therefore the binary coding was maintained to assess broadly for between-group discrepancies based on racialized experiences. Zwaigenbaum et al. (25) examined the sex differences in this cohort and found that, while a clear sex ratio was present, the ratio was lower than reported in population studies and less dependent on co-occurring cognitive or intellectual challenges. Descriptive studies such as Zwaigenbaum et al. (25) suggest that identification of children assigned female at birth may go undetected until much later in life (26) and necessitate research into exploring potential differences in females and minority phenotypes of ASD.

### 4.2 Implications and future directions

Overall, the subgroup differences in developmental trajectories, as demonstrated by trends in parental concerns, demonstrate that those diagnosed earlier (at 18 months) vs. later (at 24–36 months) in early life have phenotypic differences in their presentations of ASD. That early concerns of parents in the later identified subgroups were significantly different from those in the earlier identified subgroup highlights the heterogeneity of ASD in the early years and necessitates research into the early behavioural features of children with emerging autism even earlier in development. The findings additionally highlight the value of parent-reported early concerns, in a brief interview format, as a tool that can alert clinicians to the early features of autism, further attention to which may help reduce disparities in age of diagnosis by providing valuable information that is not as easily captured in short clinical visits. The semi-structured nature of the interview may be of particular importance, as domain-specific answers may elicit responses that are missed when exclusively using open-ended questions. Future research should aim to increase sensitivity in early diagnostic and screening tools that map onto more nuanced profiles of autism in infancy, as well as identification of factors that predict thriving and challenges later in life, allowing for more targeted interventions to be applied. Further, clinicians should note that, at a minimum within the context of infant early detection, a previous assessment in which a child does not meet criteria for ASD does not eliminate the possibility of a future diagnosis, particularly when parents describe emerging developmental differences and concerns.

## Data Availability

The raw data supporting the conclusions of this article will be made available by the authors, without undue reservation.
