# Supplementary material for: Parental concerns correspond to earliest age of autism diagnosis in increased likelihood infant cohort
Source: Front Child Adolesc Psychiatry. 2026 Jan 7;4:1722543. doi: 10.3389/frcha.2025.1722543 (PMC12819635; doi:10.3389/frcha.2025.1722543)
Supplement: Supplementary file 1 [file Datasheet1.pdf]

Parent Concern Form

Child's Name: ______________________________ **Age:** ______________ **Date:** ____________________

Growth: Height: __________cm Weight: __________kg Head Circumference: __________cm

Do you have any questions that you would like to discuss? ____

____

Do you have any concerns about current behaviour and development (including changes or loss of skills)?

____

____

HEALTH

Sleep: Are there any current concerns about sleep? (Circle one: **yes** **no**)

What is the current pattern of sleeping?

Duration: ____

Difficulties settling to sleep: ____

Night wakening (# of times, how long, methods to go back to sleep): ___________________________

____

Diet: Are there any current concerns about eating habits? (Circle one: **yes** **no**)

Describe current foods taken: ____

____

Describe concerns, including changes in eating habits/type of food taken: _______________________

____

SENSORY

Does your child have any sensitivities or sensory interests that seem unusual? (Circle one: **yes** **no**)

If yes, record examples.

Sounds: ____

Textures: ____

Visual Inspection: ____

Describe any changes since last visit: ____

DEVELOPMENT

Are there any current concerns about motor development? (Circle one: **yes** **no**)

Motor: How does your child move around and manipulate objects now?

Gross motor use/tone: ____

Fine motor use/tone: ____

Any unusual/repetitive movements? Describe: ___________________________________

____

Are there any current concerns about language development? (Circle one: **yes** **no**)

Communication: How does your child communicate?

Examples of babbling and words: ____

____

Examples of non-verbal communication (eye contact, gaze shifts, gestures): ______________

____

Communicative functions (requests/attention seeking/comments/protests): _______________

____

Has there been any loss or change in language or how your child communicates? (Circle one: **yes** **no**)

If yes, describe (and note regression on Milestone Tracking Sheet). Losses or change in:

Use of words (note maximal number of words prior to loss, timing and duration of loss, whether words have redeveloped: ____

Amount of babbling/talking: ____

Response to name with E/C: ____

Unusual sounds (age of onset): ____

Gestures: ____

Are there any concerns about how your child interacts socially? (Circle one: **yes** **no**)

If yes, describe: ____

____

____

Are there any current concerns about how your child plays? (Circle one: **yes** **no**)

Describe child’s play interests: ____

____

____

BEHAVIOUR

Are there any current concerns about your child’s behaviour or how they react to things? (Circle one: **yes** **no**)

If yes, describe concerns (note changes since last visit, soothability, irritability, transitions, anxieties): ________

____

____

____

ADDITIONAL NOTES

____

____

____

____

____

____

____

____

____

____

____
